# Membrane Binding of Parkinson's Protein α‐Synuclein: Effect of Phosphorylation at Positions 87 and 129 by the S to D Mutation Approach

**DOI:** 10.1002/ijch.201600083

**Published:** 2016-11-10

**Authors:** Pravin Kumar, Nathalie Schilderink, Vinod Subramaniam, Martina Huber

**Affiliations:** ^1^ Department of Physics, Huygens-Kamerlingh-Onnes Laboratory Leiden University Leiden The Netherlands; ^2^ Nanobiophysics, MESA+ Institute for Nanotechnology University of Twente Enschede The Netherlands; ^3^ FOM Institute AMOLF Amsterdam The Netherlands; ^4^ Vrije Universiteit of Amsterdam Amsterdam The Netherlands

**Keywords:** alpha synuclein, phosphorylation, membrane binding, EPR spectroscopy

## Abstract

Human α‐synuclein, a protein relevant in the brain with so‐far unknown function, plays an important role in Parkinson's disease. The phosphorylation state of αS was related to the disease, prompting interest in this process. The presumed physiological function and the disease action of αS involves membrane interaction. Here, we study the effect of phosphorylation at positions 87 and 129, mimicked by the mutations S87A, S129A (nonphosphorylated) and S87D, S129D (phosphorylated) on membrane binding. Local binding is detected by spin‐label continuous‐wave electron paramagnetic resonance. For S87A/D, six positions (27, 56, 63, 69, 76, and 90) are probed; and for S129A/D, three (27, 56, and 69). Binding to large unilamellar vesicles of 100 nm diameter of 1‐palmitoyl‐2‐oleoyl‐*sn*‐glycero‐3‐phospho‐(1′‐rac‐glycerol) and 1‐palmitoyl‐2‐oleoyl‐*sn*‐glycero‐3‐phosphocholine in a 1 : 1 composition is not affected by the phosphorylation state of S129. For phosphorylation at S87, local unbinding of αS from the membrane is observed. We speculate that modulating the local membrane affinity by phosphorylation could tune the way αS interacts with different membranes; for example, tuning its membrane fusion activity.

## 1 Introduction

**Figure 1:**
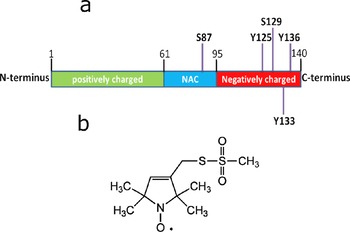
a) The most common phosphorylation sites in αS. Given is the sequence number (in boldface), preceded by the residue (Y or S) that is phosphorylated. Also, the important regions of the protein are shown, indicated by sequence numbers at the start and end. Positively charged (green): net positive charge of protein between residues 1 and 61; NAC (blue): non‐amyloid‐β component; and negatively charged C‐terminal part of the protein (red), from residue 95 onwards. b) Chemical structure of the spin‐label MTSL, by which cys is labelled.

Parkinson's disease^1^ is the second‐most prevalent neurodegenerative disease after Alzheimer's disease.^2^ This disease is characterized by the formation of protein deposits, such as Lewy bodies, in the brain.^3^,^4^ The protein α‐synuclein (αS) constitutes the main component of these deposits.^5^, ^6^, ^7^ A number of posttranslational modifications of αS are present within the Lewy bodies in Parkinson's disease (PD) and related disorders.^8^,^9^ The major disease‐associated posttranslational modifications are phosphorylation,^8^,^10^ truncation, ubiquitination,^11^ and also oxidation (like nitration),^12^ but one of the key posttranslational modifications is phosphorylation. The protein αS has been found hyperphosphorylated in Lewy bodies and Lewy neurites.^1^,^9^,^13^ The role of phosphorylation of αS in neurotoxicity is controversial. However, growing evidence suggests that phosphorylation could influence membrane/vesicle binding of αS and its aggregation.^8^,^14^, ^15^, ^16^, ^17^ Recent reviews summarize results of *in vivo* and *in vitro* studies performed up to now and describe to which degree phosphorylation of αS is linked to disease.^18^,^19^ The major phosphorylation sites of αS are shown in Figure 1. The phosphorylation sites Y125, S129, Y133, and Y136 are the most discussed in the literature; for example, S129 is highly phosphorylated in Lewy bodies. One more phosphorylation site, S87, is special, since it distinguishes the human αS sequence from that of mouse and rat. Also, a link between phosphorylation at 87 and disease was discussed by Paleologou *et al*.^13^

Here, we focus on the membrane‐binding aspect of αS phosphorylation *in vitro* at positions S87 and S129. Membrane binding of αS concerns an amphipathic helix spanning residues 1–100.^20^, ^21^, ^22^ The N‐terminal half (residues 1–50) of the amphipathic helix is termed helix 1, and the other half (residues 51–100), helix 2. The affinity of αS to membranes depends on the negative charge density (ρ) of the membrane, where ρ represents the molar fraction of anionic lipids present in the membrane.^23^ Different binding properties were found for helix 1 and helix 2.^24^

There are three ways to generate protein constructs to study the effect of phosphorylation: 1) to phosphorylate the respective residues enzymatically, which requires dedicated enzymes/overexpression systems^25^,^26^ and is reversible; 2) by a semisynthetic approach, in which a (phosphorylated) peptide is linked to the corresponding overexpressed protein;^27^ and 3) by generating mutants whose side chains mimic the chemical properties of the phosphorylated state (negative charge) and size, sometimes referred to as pseudophosphorylation.^28^ Typically, S is replaced by D or E^13^,^17^,^29^,^30^ to mimic phosphorylation, and alanine is used as the reference for the nonphosphorylated state, especially for *in vivo* studies.

All three approaches have been used to study αS phosphorylation *in vivo* and *in vitro*, showing that in some cases, enzymatically phosphorylated αS (P‐αS) and pseudophosphorylated αS can behave differently.^29^,^31^ For example, enzymatic phosphorylation of αS at S129 has been shown to have an inhibitory effect on αS aggregation, while pseudophosphorylation does not show such an effect.^29^ Apparently, the different behavior depends strongly on the properties probed and the environment αS is exposed to. In the present study, we focus on the phosphomimic approach with the S→D substitution to mimic phosphorylation, and investigate the constructs S87A or S129A (nonphosporylated); and S87D or S129D (phosphorylated).

We used large unilamellar vesicles (LUVs) as membrane models with a 1 : 1 mixture of the lipids 1‐palmitoyl‐2‐oleoyl‐*sn*‐glycero‐3‐phospho‐(1′‐rac‐glycerol) (POPG) and 1‐palmitoyl‐2‐oleoyl‐*sn*‐glycero‐3‐phosphocholine (POPC), generating a membrane with a charge density ρ=0.5. Previous studies on model membranes showed that at high charge densities, i.e., above 0.8–0.9, αS is fully bound to those membranes,^23^,^24^,^32^, ^33^, ^34^ revealing that the interaction is strong and dominated by electrostatics, which risks masking the effects of phosphorylation. Additionally, such charge densities are nonphysiological, so we avoided these high negative charge densities. At low charge densities (ρ≤0.2), i.e., on neutral or weakly negatively charged membranes, binding is very low, resulting in a large fraction of unbound protein, which would also abolish any differential binding effect of phosphorylation. This made ρ=0.5 an optimum charge density at which to work.

To investigate membrane binding, we used spin‐label electron paramagnetic resonance (EPR) spectroscopy. For spin labelling, the amino‐acid residue at the sequence position of interest is replaced by a cysteine, which is reacted with a suitable functional group of the nitroxide spin label (see Figure 1b), an approach introduced by the Hubbell group.35a In this way, a nitroxide, which contains an unpaired electron and is therefore EPR active, is covalently attached to the protein. Then the properties of the protein can be probed at the modified position by EPR. In the present study, we make use of the ability of EPR to detect the mobility of the spin label by room‐temperature continuous‐wave (cw) EPR. Characteristic line shapes of the spectra reveal the mobility of the spin label, with narrow lines corresponding to fast motion (i.e., rotational correlation times (*τ_r_*) of several hundreds of ps) and broad lines to slow motion, in the ns‐regime. In our particular case, slow motion of the spin label shows that the section of the protein to which the spin label is attached is bound to the membrane, whereas fast motion shows detachment of the protein from the membrane. The methodology described was introduced before and has proven valuable for determining the local binding of αS to membranes.^24^,^32^, ^33^, ^34^

The spin‐labelled constructs are referred to as *SLposition*αS/S87A(D) or *SLposition*αS/S129A(D), such that, for example, SL27αS/S87D is the construct with the spin label at position 27 and is the phosphorylated variant at position 87. We investigated several spin‐label positions for each phosphorylation site, resulting in a total of nine constructs, as summarized in Table 1.

**Table 1:** The αS constructs used to study phosphorylation at position S87 and S129; SL denotes the position of the spin label.

Spin‐label positions	S87A (nonphosphorylated)	S87D (phosphorylated)
SL27	SL27αS/S87A	SL27αS/S87D
SL56	SL56αS/S87A	SL56αS/S87D
SL63	SL63αS/S87A	SL63αS/S87D
SL69	SL69αS/S87A	SL69αS/S87D
SL76	SL76αS/S87A	SL76αS/S87D
SL90	SL90αS/S87A	SL90αS/S87D

Spin‐label positions	S129A (nonphosphorylated)	S129D (phosphorylated)
SL27	SL27αS/S129A	SL27αS/S129D
SL56	SL56αS/S129A	SL56αS/S129D
SL69	SL69αS/S129A	SL69αS/S129D

In this work, we show how phosphorylation affects the binding of αS to the membrane. It decreases the binding of αS to the membrane when phosphorylated at the S87 position, whereas no effect is seen when phosphorylated at the S129 position. We also show that phosphorylation at position 87 does not detach the protein completely from the membrane, but rather, causes local unbinding, which is particularly pronounced in the helix 2 region.

## 2 Results and Discussion

### 2.1 Results

We investigate the binding of phosphorylation variants of αS at positions 87 and 129 to LUVs of 100 nm diameter. The LUVs are composed of a 1 : 1 mixture of POPG and POPC, generating a membrane of charge density ρ=0.5. We first describe the results of phosphorylation at position 87, then at 129.

**Figure 2:**
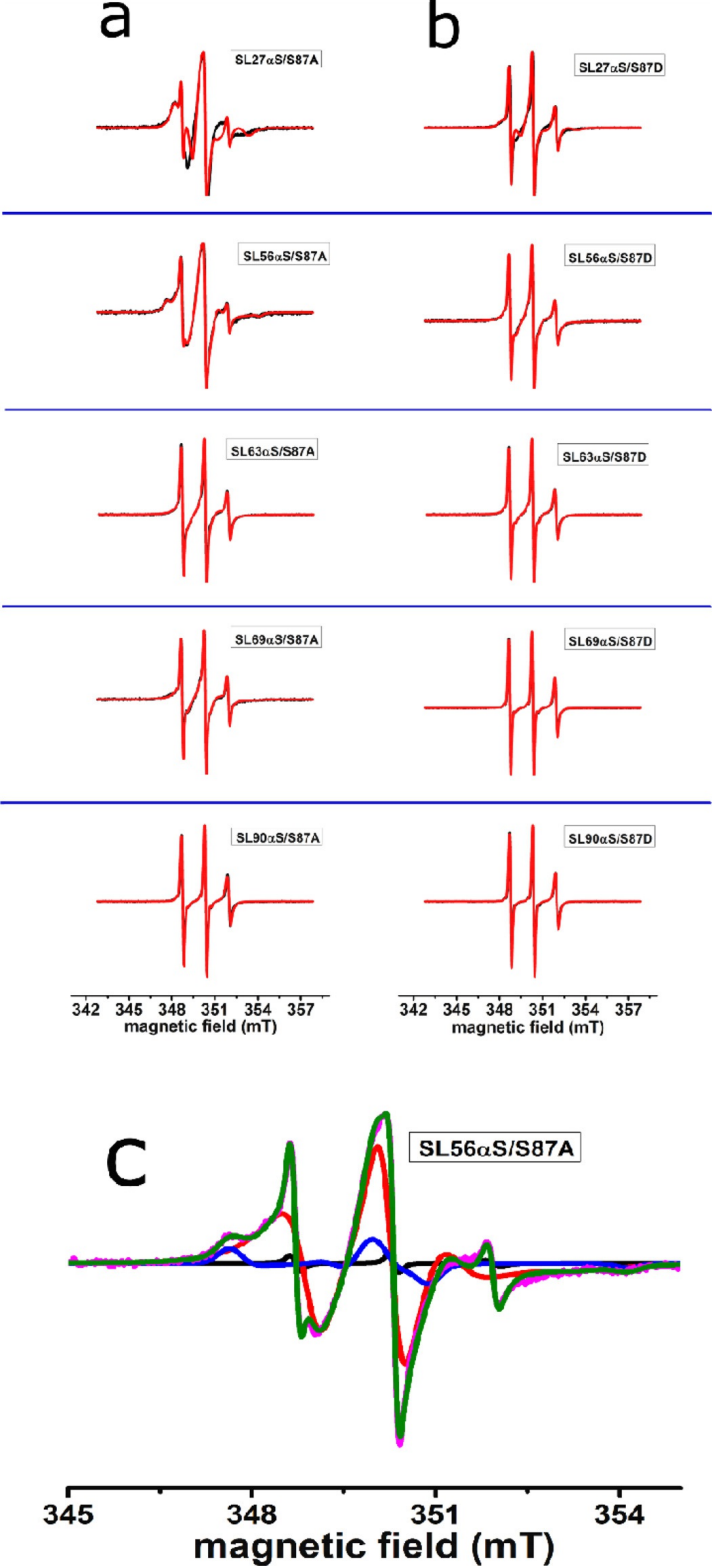
Effect of phosphorylation at position 87 on αS binding to LUVs: room‐temperature EPR spectra of spin‐labelled αS constructs (for nomenclature see Table 1) with LUVs of a 1 : 1 mixture of POPG and POPC: a) nonphosphorylated; and b) phosphorylated forms. Black line: experiment; red line: simulation. c) Decomposition of EPR spectrum into components shown for SL56αS/S87A. The fast (black), slow (red), and immobilized (blue) components are shown, as well as the added simulation (green) and the experimental spectrum (pink).

**Figure 3:**
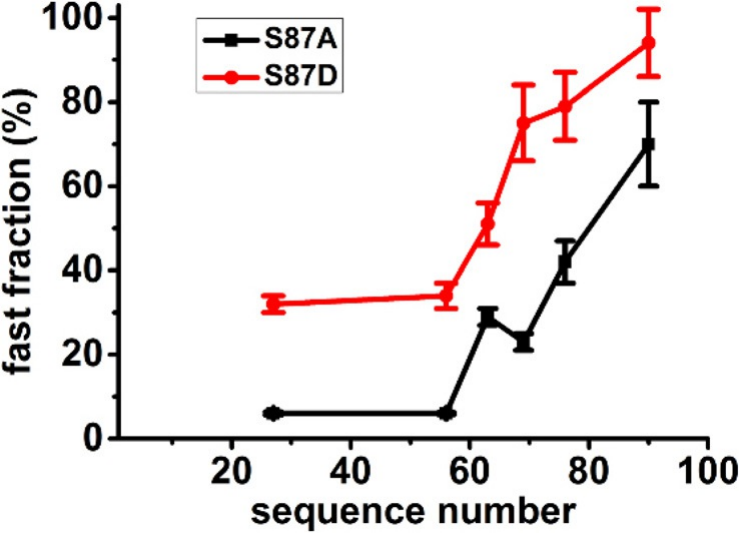
Local‐unbinding effect of phosphorylation at position 87: amount of fast fraction in αS 87 A,D mutants in the presence of LUVs as a function of the sequence number. Black: nonphosphorylated (αS87A); red: phosphorylated (αS87D) (see Table 2 for values); the lines connecting the points are guides for the eye.

Figure 2 shows the spectra of the spin‐labelled constructs probing phosphorylation at position 87 in the presence of LUVs (for the complete list of constructs, see Table 1). In this set, helix 1 is probed in the middle, at residue 27; helix 2 is probed at five probing positions, starting from position 56 and terminating at 90. Figure 2a shows the spectra of αS in the nonphosphorylated form and Figure 2b in the phosphorylated form. The spectra in Figure 2a differ from those in Figure 2b; most notably, each spectrum in Figure 2b has narrower lines than its counterpart in Figure 2a. As described in the introduction, narrow lines derive from spin labels that are rotating fast. As discussed in more detail below, fast rotation shows that the section of the protein to which the spin label is attached is not bound to the membrane. More detailed information was obtained by spectral simulation of the experimental spectra, which yields the parameters of mobility of the spin label, the rotational correlation time (*τ_r_*), and in the case of multicomponent spectra, the amount by which each fraction contributes. These parameters are given in Table 2. In Figure 2c, an example of a simulation is shown. Three fractions are visible: the fast, the slow, and the immobile components, which have increasingly large linewidths. The individual components add up to give the experimental spectrum. Table 2 reveals that all but two spectra consist of a superposition of two components, the fast and slow components, except for the SL56αS/S87A variant, which, in addition, has a third, the immobile component, and the SL90αS/S87A and SL90αS/S87D variants, which have only one component, the fast component. Each component reflects a part of the protein population: the fast fraction is due to protein in which the region around the site that is spin labelled is not attached to the membrane, whereas the slow and immobilized fractions are due to the sections bound to the membrane. The amount by which each component contributes to the spectra (Table 2, columns four and six) reflects the fraction of protein contributing to each component. The correlation times can be determined to several tens of ps in the case of the fast fraction, and several hundred ps for the slow fraction (see Table 2). The contribution of the fast component of αS in the nonphosphorylated form is smaller than in the phosphorylated form for each probing position. The opposite is the case for the contribution of the slow components. Both these trends reveal that phosphorylation reduces membrane binding. To illustrate the effect of phosphorylation at position 87, Figure 3 shows a plot of the amount of the fast fraction for phosphorylation at position 87 as a function of the sequence number at which mobility is probed.

**Table 2:** Effect of phosphorylation of αS at position 87 (S87A/D): parameters describing the mobility of the spin label in the EPR spectra; *τ_r_* is the rotation correlation time of the spin label.

αS spin‐label positions	Components contributing to spectra	S87A (nonphosphorylated)		S87D (phosphorylated)	
		*τ_r_* (ns)	Contribution (%)	*τ_r_* (ns)	Contribution (%)
SL 27	fast	0.4±0.03	6±0.4	0.4±0.02	32±2
	slow	8.5±0.2	94±0.4	9.3±0.65	67±2
	immobile	na	na	na	na
SL 56	fast	0.4±0.02	6±0.2	0.4±0.03	34±3
	slow	3.2±0.07	78±1.2	3.1±0.4	63±3
	immobile	>50	16±1	na	na
SL 63	fast	0.35±0.04	29±2	0.4±0.03	51±5
	slow	2.6±0.3	70±2	2.5±0.6	44±5
	immobile	na	na	na	na
SL 69	fast	0.3±0.02	23±2	0.3±0.02	75±9
	slow	2.5±0.2	75±2	2.5±1.2	20±9
	immobile	na	na	na	na
SL 76	fast	0.4±0.04	42±5	0.4±0.02	79±8
	slow	3.5±0.8	57±5	3.5±3.2	16±8
	immobile	na	na	na	na
SL 90	fast	0.4±0.04	70±10	0.3±0.03	100^[a]^±8
	slow	2.5±1.3	24±10	na	na
	immobile	na	na	na	na

na: a component seen in other spectra, but not required to obtain a good simulation of the experimental spectrum in question, revealing that the rotational correlation time of the spin label does not contain contributions on the timescale of the component in question (for details, see text and Figure 2). For error determination, see Section 4. [a] including 4.5 % contribution of spin label with natural abundance of ^13^C.

For all monitoring positions, the amount of the mobile fraction is larger in the phosphorylated variant. At monitoring positions 27 and 56, the amount of mobile fractions of nonphosphorylated αS is below 10 %, which indicates strong binding, but at later positions (helix 2), the amount of fast fractions increases to 70 %, indicating the loosening of the helix 2 of αS when it is nonphosphorylated, in agreement with previous findings for wt αS.^24^ For the phosphorylated αS, the amount of the mobile fraction is higher than in the nonphosphorylated form for all positions monitored, enhancing the tendency for local unbinding in helix 2, until, at position 90, the bound fraction is so low that it becomes undetectable.

To determine if the phosphorylation reduces the overall membrane affinity of αS, i.e., if αS detaches completely from the membrane, resulting in αS protein that is free in solution (physical unbinding), we separated the physically unbound fraction of αS from the membrane‐bound fraction, by filtrating the sample through a filter that retains the vesicles and αS bound to them. The amount of physically unbound protein in the filtrate is then determined by EPR, as described in Drescher *et al*.^24^ (for details, see Section 4). The amount of unbound αS is given in Table 3, and is below 16 % for all constructs. Thus, the amount of physically unbound αS is significantly lower than the amount of the fast fraction measured by EPR (see Table 2), showing that the local unbinding far outweighs any physical unbinding. The percentages in Table 3 for spin‐label positions 27 and 56 are slightly lower than for the other positions. Given that the differences are just outside the error margins of the procedure, we cannot draw conclusions.

**Table 3:** Physical unbinding of αS S87D from the membrane: results of filtration experiments (for details, see Sections 4 and 2.1).

mutants	αS unbound fraction (%)
SL27αS/S87D	5.9±2
SL56αS/S87D	5.2±1
SL69αS/S87D	15.1±3
SL90αS/S87D	13.6±3

**Figure 4:**
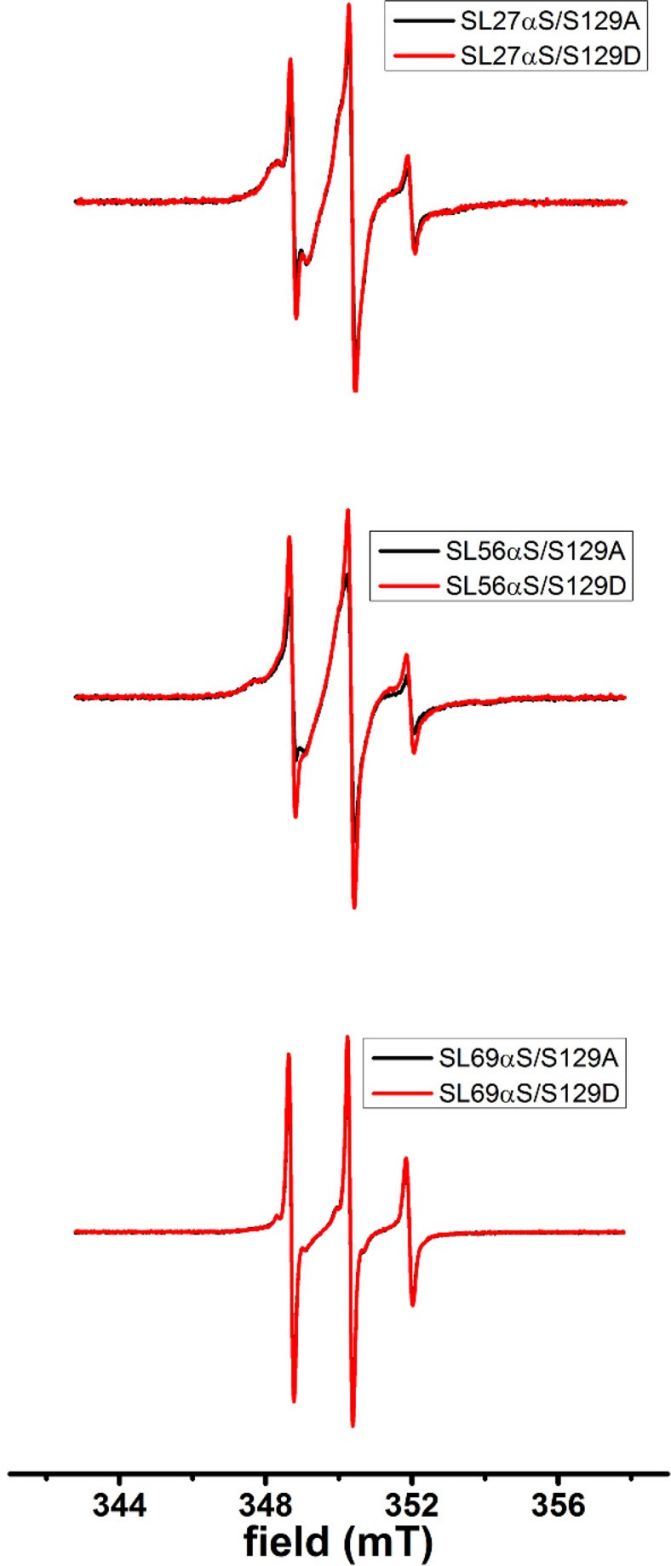
Effect of phosphorylation at position 129 on αS binding to LUVs: room‐temperature EPR spectra of spin‐labelled αS constructs (for nomenclature, see Table 1) with LUVs of a 1 : 1 mixture of POPG and POPC; superposition of nonphosphorylated (black line) with phosphorylated EPR spectra (red line), normalized by their double‐integral value.

For phosphorylation at position 129, Figure 4 shows the superposition of the spectra of nonphosphorylated and phosphorylated variants for three spin‐label positions (see Table 1). In contrast to phosphorylation at position 87, A and D variants at position 129 have similar spectra, obviating the need for detailed spectral analysis. Apparently, phosphorylation has a much smaller influence at position 129 than at position 87.

### 2.2 Discussion

We have investigated how the membrane binding of αS depends on the phosphorylation state of positions 87 and 129. Membrane binding is detected locally, via the mobility of spin labels attached to specific positions in the protein. An increased spin‐label mobility shows that the protein detaches from the membrane around the position probed.

The membrane composition was chosen to be conducive to intermediate binding, with a charge density of ρ=0.5, to avoid dominant electrostatic effects, which are observed at higher charge densities, where they cause strong, undifferentiated binding and are nonphysiological, or low charge densities, causing overall unbinding,^23^,^24^,^32^, ^33^, ^34^ as described in the Introduction. The membrane was offered in the form of LUVs of a diameter of 100 nm. We mimic phosphorylation by the phosphorylation‐mutation approach, replacing S by D, an approach used before^13^,^17^,^29^,^30^ (for details, see Introduction). Although some studies showed that biochemically phosphorylated αS can have different properties than phosphorylation mimics,^29^,^31^ the latter constructs provide a robust system to study phosphorylation effects *in vitro*, explaining their popularity.

Under the conditions of our study, phosphorylation at position 129 has no noticeable effect on membrane binding, whereas 87 has, similar to what was observed by other techniques in the past.^13^ In the following, we will first discuss the influence of phosphorylation at position 87 on αS membrane binding, and then compare the results obtained on both phosphorylation sites to previous findings in the literature.

When position 87 is phosphorylated, membrane binding is reduced relative to the nonphosphorylated case. An almost constant reduction of the binding is observed at positions 27 and 56 in the helix 1 region: see Figure 3. Similar to wild‐type αS,^24^ also in the S87A variants, helix 2 has a lower membrane affinity than helix 1. Phosphorylation enhances this trend, up to the point that at probing position 90, the bound fraction becomes so low that is undetectable within experimental error. Complete physical detachment of the phosphorylated protein from the membrane does not play a role: as seen in Table 3, the physically unbound fraction is below 16 % for all constructs. To place this into perspective, the amount of physically unbound αS is maximally one‐third of the amount of fast fraction determined from EPR, showing that the majority of the fraction, seen by EPR, derives from protein that is attached to the membrane, presumably at the residues preceding the probed sequence position, e.g., for sample SL27/αS87P, residues 27 and below. Fluctuations in the amount of fast fraction (Table 2, SL 63, nonphosphorylated (SL63/S87A) has a larger amount of fast fraction than SL 69), and a larger amount of physically unbound αS for SL positions in helix 2 (Table 3), could indicate an influence of the spin label on αS membrane binding. If such an effect is present, it never exceeds a contribution of 10 %, and therefore is not relevant for the conclusions drawn.

Overall, we find that phosphorylation at position 87 decreases the membrane affinity of αS, particularly for helix 2. This effect is fully consistent with the change in the charge caused by the conversion of S→D or by phosphorylation: A negative charge in the helix 2 will weaken the electrostatic interaction with the negatively charged membrane surface, as it counteracts the effect of several lysines (Lys; K) in the αS sequence from residues 1–100. Reduced membrane binding of S87E and P‐S87 has been reported before, e.g., Refs. 13 and 35b.

Reduced membrane binding affects the entire protein, but is most pronounced in the helix 2 region, and may selectively influence the behavior of helix 2. Some models propose that the physiological function of αS involves vesicle fusion events in which helix 1 and helix 2 interact with different types of membranes.^36^ We therefore speculate that phosphorylation at position 87 could be used to tune how αS operates in vesicle trafficking.

For the αS129 A/D variants, the difference in mobility of the spin label for phosphorylated and nonphosphorylated forms is minute, showing that under the membrane conditions employed here, phosphorylation at this site does not affect membrane binding. The C‐terminus of αS is already negatively charged and was not found to interact with the membrane in previous studies,^20^,^21^,^24^,^37^,^38^ which is fully consistent with the lack of changes in membrane binding observed in the present study upon phosphorylation at position 129.

The results of the present study suggest that phosphorylation at position 87 tunes those functions of αS that involve membrane binding and vesicle interaction, whereas phosphorylation at position 129 acts on other aspects of αS in the organism. Previously,^13^ several possibilities of how phosphorylation at 129 could affect αS *in vivo* behavior have been described and the study of Kosten *et al*.^39^ shows that the phosphorylation at position 129 depends on the phosphorylation state of position 125, suggesting a complex interplay of posttranslational modifications in the C‐terminus.

Most of the current research is focused on phosphorylation at position 129, and the phosphorylation degree at this position is related to disease effects, as reviewed in Ref. 40. In agreement with our results, several studies show that αS phosphorylation at 129 has no or little effect on membrane binding; see, for example, Ref. 28; however, several studies find an influence of phosphorylation at 129 on the aggregation of αS^28^,^29^,^41^ and on membrane binding of αS aggregates,^41^ suggesting that *in vivo* effects are linked to aggregation‐sensitive processes.

## 3 Conclusion

In conclusion, the large spectrum of phosphorylation effects on αS *in vivo* and *in vitro* 13,14,16,19,28, 29, 30, 31,35b,^40^, ^41^, ^42^, ^43^, ^44^, ^45^, ^46^, ^47^, ^48^, ^49^, ^50^ furnishes the need for isolating the different factors that can be modulated by αS phosphorylation *in vitro*. The present study gives one such example, where we show that *in vitro* phosphorylation mimics at position 87 (S87D) reduce αS membrane binding in a local, sequence‐dependent manner, whereas the same modification at position 129 (S129D) has no influence on membrane binding. We expect that this approach provides a foothold to interpreting the challenging *in vivo* physiological and pathological functions of αS.

## 4 Materials and Methods

### 4.1 Protein Expression and Labelling

All αS mutants were expressed in *Escherichia coli* strain BL21(DE3) using the pT7‐7 expression plasmid and purified in the presence of 1 mM DTT, as previously reported^51^,^52^ Serine‐87 is substituted either by Alanine (S87A, represents phosphorylation‐ inactive form) or by Aspartate (S87D, represents phosphomimic form). For labelling, a cysteine mutation was introduced at the desired residues.

Spin labelling was done following the standard protocol, described briefly. Before starting labelling, αS cysteine mutants were reduced with a six‐fold molar excess per cysteine with DTT (1,4‐dithio‐D‐threitol) for 30 min at room temperature. To remove DTT, samples were passed through a Pierce Zeba 5 ml desalting column. Immediately, a ten‐fold molar excess of the MTSL spin label ((1‐oxyl‐2,2,5,5‐tetramethylpyrroline‐3‐methyl)‐methanethiosulfonate) was added (from a 25 mM stock in DMSO) and incubated for 1 h in the dark at room temperature. After this, the free spin label was removed by using two additional desalting steps. Protein samples were applied onto Microcon YM‐100 spin columns to remove any precipitated and/or oligomerised proteins and were diluted in buffer (10 mM Tris‐HCl, pH 7.4). Spin‐label concentrations were 2.5 mM at protein concentrations of 250 μM. Owing to the high reactivity of the label and the fact that the cysteine residues were freely accessible in the poorly folded structure, near quantitative labelling could be achieved under these conditions.^37^ Samples were stored at −80 °C.

### 4.2 Preparation of Vesicles

All lipids were purchased from Avanti Polar Lipids, Inc. as chloroform solutions and were used without further purification. LUVs were prepared from 1 : 1 mixtures of 1‐palmitoyl‐2‐oleoyl‐*sn*‐glycero‐3‐phospho‐(1′‐rac‐glycerol) (POPG) and 1‐palmitoyl‐2‐oleoyl‐*sn*‐glycero‐3‐phosphocholine (POPC). Lipids were mixed in the desired ratio and then chloroform was evaporated by dry nitrogen gas. The resulting lipid films were kept under vacuum overnight. Dried lipid films were hydrated with 10 mM Tris‐HCl, pH 7.4 for 1 hour at 30 °C, and the resulting milky lipid suspensions were extruded through 100 nm pore size polycarbonated membranes using the mini extruder (catalogue no. 610000) from Avanti Polar Lipids. The size of the vesicles was determined by dynamic light scattering (DLS). The DLS‐experiments were performed on a Zetasizer Nano‐ZS (Malvern). We obtained vesicles with a homogeneous size distribution around *d*=100 nm.

### 4.3 Sample Preparation

Spin‐labelled αS mutants were added from stock solutions (concentration between 150 μM and 250 μM) to the LUVs to obtain a lipid to protein ratio (L : P) of 250 : 1, and incubated for 30 min at room temperature before measuring. All samples were prepared and measured at least three times. All spin‐labelled αS constructs used in this work are shown in the Table 1.

### 4.4 Filtration Experiments

To determine, if αS physically detaches from the membrane, we performed filtration experiments similar to those described in Drescher *et al*.^24^ An αS vesicle solution, prepared as for the EPR experiments described above (sample preparation), was passed through a 100 kDa cutoff filter device (Amicon Ultra 100k) which retained the vesicles, and thereby, the membrane‐bound αS fraction, but was permeable for unbound αS. The concentration of αS in the filtrate was too low to measure directly; therefore, the filtrate was concentrated using a 3 kDa cutoff filter device (Amicon Ultra 3k) and measured by EPR to determine the amount of αS in the filtrate. The error in the final value, of the order of 20 %, is largely due to the errors in determining the volumes before and after the concentration step, and the error of the double‐integral procedure to determine the spin concentration by EPR.

### 4.5 Continuous‐wave EPR Experiments

The X‐band continuous‐wave EPR measurements were performed using an ELEXSYS E680 spectrometer (Bruker, Rheinstetten, Germany) with a super high Q cavity (ER 4122 SHQE‐W1/1108). Measurements were performed at 20 °C, using 0.63 mW of microwave power, 100 kHz modulation frequency, and a modulation amplitude of 1.0 G. Total acquisition time for the EPR spectra was 20 minutes.

### 4.6 Simulation of cw‐EPR Spectra

Spectral simulations were performed using Matlab (7.11.0.584, Natick, Massachusetts, U.S.A.) and the EasySpin package.^53^ For all simulations, the following spectral parameters were used: *g*=[2.00906, 2.00687, 2.00300]^54^ and the hyperfine tensor parameters *A*_*XX*=_ *A*_*YY*=_13 MHz. Usually, a superposition of more than one component was required to simulate the spectra. The parameters were manually changed to check in which range acceptable simulations of the experimental spectra were obtained to determine the error margins. To simulate spectra of αS bound to membranes, the *τ_r_* of the fastest component was kept at the *τ_r_* value of the spectra of the respective protein construct, in the absence of vesicles.
